# Myocardial perfusion MRI compared to fractional flow reserve: a meta-analysis

**DOI:** 10.1186/1532-429X-17-S1-P231

**Published:** 2015-02-03

**Authors:** Richard Takx, Björn Blomberg, Pim A de Jong, Tim Leiner

**Affiliations:** Radiology, UMC Utrecht, Utrecht, Netherlands; Radiology, MGH, Boston, MA USA

## Background

Myocardial perfusion MRI is a technique that allows for detection myocardial ischemia. The goal of this meta-analysis is to determine the diagnostic accuracy of Myocardial perfusion MRI compared to fractional flow reserve (FFR) for the diagnosis of hemodynamically significant coronary artery stenosis.

## Methods

This meta-analysis was performed in adherence to the PRISMA statement. Two reviewers systematically searched PubMed, EMBASE and Web of Science, using predefined inclusion and exclusion criteria. Only studies using invasive coronary angiography combined with FFR for assessment of intermediate coronary stenoses were included. The QUADAS-2 criteria were applied for quality appraisal. A random-effects model was used for computing pooled sensitivity, specificity, likelihood ratios, and the diagnostic odds ratio. Analyses were performed on both vessel and patient levels.

## Results

In total, 15 studies (1830 vessels/798 patients) satisfied the predefined inclusion criteria. At the vessel level MRI performed very good with a pooled sensitivity of 0.87 (95% CI 0.84, 0.90) and a pooled specificity of 0.91 (95% CI 0.89, 0.92). At patient level the results were similar with a pooled sensitivity of 0.89 (95% CI 0.86, 0.92) and a pooled specificity of 0.87 (95% CI 0.83, 0.90).

## Conclusions

Myocardial perfusion MRI is a highly accurate technique to detect flow limiting coronary stenosis and has the potential to function as a gatekeeper for invasive angiography.

